# Strategies in immune subversion: How *Yersinia pestis* inhibits neutrophil responses

**DOI:** 10.1371/journal.ppat.1014120

**Published:** 2026-04-10

**Authors:** Katelyn R. Sheneman, Matthew B. Lawrenz

**Affiliations:** 1 Department of Microbiology and Immunology, University of Louisville School of Medicine, Louisville, Kentucky, United States of America; 2 Center for Predictive Medicine for Biodefense and Emerging Infectious Diseases, University of Louisville School of Medicine, Louisville, Kentucky, United States of America; University of Geneva: Universite de Geneve, SWITZERLAND

The dermis and mucosal surfaces (e.g., the lungs and gastrointestinal tract) are primary sites of mammalian interactions with pathogens and key physical and immunological barriers to infection. Neutrophils serve as a critical first line of immunological defense in these tissues. These cells employ a multitude of mechanisms to limit the proliferation and dissemination of pathogens. At the forefront are antimicrobial responses that directly contribute to the killing of microbes. These include phagocytosis, the generation of reactive oxygen species (ROS), and the release of antimicrobial proteins through degranulation. Moreover, neutrophils are also key to initiating the inflammatory cascade through the release of inflammatory lipids and proteins (e.g., cytokines and chemokines) that prime and recruit innate and adaptive immune cells to respond to the infection. Together, these mechanisms typically limit microbial colonization, proliferation, and dissemination. However, *Yersinia pestis*, which causes human plague, and the related species *Y. pseudotuberculosis* and *Y. enterocolitica* have evolved mechanisms to actively repress the ability of neutrophils to control infection. Moreover, rapid evasion of neutrophils in the dermis/lungs or the gastrointestinal tissues are essential for *Y. pestis* or *Y. pseudotuberculosis* and *Y. enterocolitica*, respectively, to colonize the mammalian host. Key to immune evasion is the bacterial Type III Secretion System (T3SS) by which the bacteria translocate bacterial effector proteins—Yops—directly into host cells [1]. These Yop effectors target specific host factors to work cooperatively to inhibit signaling pathways required for neutrophil function. In this Pearl, we will highlight the molecular mechanisms used by *Y. pestis* to actively inhibit five paramount neutrophil functions (Fig 1), allowing the bacteria to evade neutrophil clearance and cause lethal infection.

**Fig 1:**
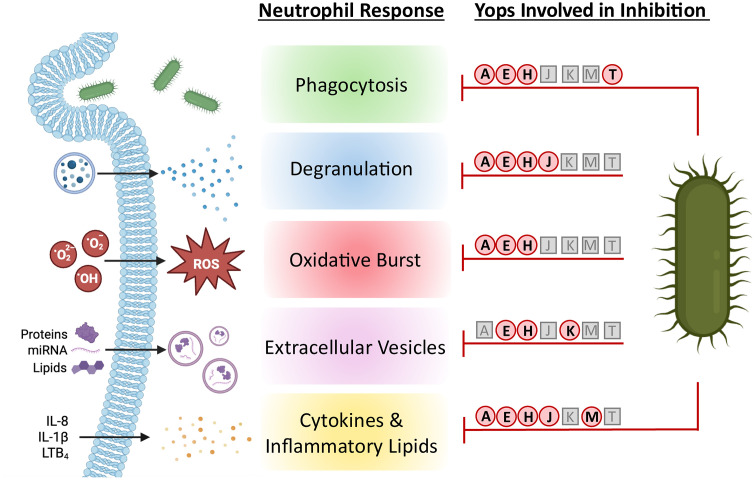
Inhibition of neutrophil effector functions by *Yersinia pestis.* Neutrophils respond in a variety of ways during infection to limit microbial growth and stimulate the immune response. However, *Y. pestis* has evolved virulence mechanisms that manipulate and inhibit these key neutrophil functions, allowing the bacteria to persist in the extracellular milieu. The T3SS Yop effectors required for inhibition of indicated neutrophil functions are represented red circles. Yop effectors that are not involved in suppression of a given neutrophil function are represented as gray squares. Created in BioRender. Lawrenz, M. (2026) https://BioRender.com/jupcia9.

## Inhibition of phagocytosis

Phagocytosis is an actin-mediated process in which neutrophils engulf and endocytose microbes into intracellular compartments called phagosomes. Microbe containing phagosomes will fuse with granule compartments, exposing the microbes to antimicrobials. However, during neutrophil interactions with *Y. pestis*, the bacteria inhibit phagocytosis in a T3SS-dependent manner. Several Yop effectors target Rho/Rac family GTPases to disrupt actin polymerization and in turn neutrophil phagocytosis and granule fusion. YopE is a GTPase-activating protein (GAP) that accelerates the hydrolysis of GTP to GDP in RhoA, Rac1/2, and RhoG to render them functionally inactive and thereby preventing the cytoskeletal rearrangements needed for phagocytosis [1–3]. As a cysteine protease, YopT actively cleaves membrane-bound Rho GTPases, disrupting the proper localization needed for these proteins to initiate the formation of the phagocytic cup [4,5]. Additionally, YpkA is a Serine/Threonine kinase that phosphorylates and subsequently inactivates a number of actin-binding proteins, inhibiting Fc-mediated phagocytosis [1,6]. YpkA also contains a guanine nucleotide dissociation inhibitor (GDI)-like domain, which allows it to sequester Rho/Rac GTPases to limit their activity [7]. In parallel to targeting host GTPases, YopH functions as a tyrosine phosphatase to disrupt receptor-mediated phagocytosis by dephosphorylating key molecules, such as PRAM-1 and SLP-76, that are downstream of Fc and GCP receptors [8]. Dephosphorylation of these proteins blunts the phosphorelay cascade required to stimulate the changes in actin dynamics needed to initiate phagocytosis. The functional redundancy of these effectors indicate that evasion of phagocytosis by neutrophils is a key virulence strategy of the bacteria.

## Suppression of reactive oxygen species

Assembly of the NADPH oxidase complex, composed of gp91^phox^, p22^phox^, p40^phox^, p47^phox^, and p67^phox^ on the plasma and phagosomal membranes, leads to the generation of ROS. ROS cause significant DNA damage, protein oxidation and lipid peroxidation that are highly toxic for bacteria. Assembly of the NADPH oxidase complex is tightly regulated and dependent on receptor engagement, calcium signaling, and cytoskeletal remodeling [9]. GPCR, integrin, or Fc receptor engagement leads to phosphorylation of Syk, Vav, and PLCγ, which in turn activate PKC, and is required for the phosphorylation of p47^phox^ and activation of Rac2. Phosphorylated p47^phox^ and activated Rac2 translocate to the membrane to stabilize the NADPH complex. Two Yop effectors have been implicated in *Y. pestis* to significantly inhibit ROS production by neutrophils—YopH and YopE. YopH inhibits ROS production in both macrophages and murine neutrophils by dephosphorylating Syk, Vav, and PLCγ to inhibit calcium flux and PKC and Rac2 activation [8,10,11]. As mentioned above, YopE also directly inactivates Rac2 via its GAP activity, and is sufficient to inhibit ROS production in the HL-60 neutrophil-like cell line [12]. Inhibition of ROS generation lowers neutrophil bactericidal activity by ≥ 50%, significantly improving the viability of *Y. pestis* in the presence of human neutrophils [13].

## Disruption of neutrophil degranulation

Neutrophils harbor four classes of granules, which are vesicular compartments densely packed with proteases, cytotoxic proteins, and antimicrobial peptides [14]. During infection, neutrophil granules quickly mobilize and fuse with the plasma membrane, expelling granule contents into the extracellular milieu, a process referred to as degranulation. Several studies have demonstrated T3SS-dependent inhibition of neutrophil degranulation by *Y. pestis* [15,16], but no single Yop effector is sufficient to inhibit degranulation. Instead, the cooperative actions of multiple Yop proteins targeting different signaling pathways are required to completely block granule release. Specifically, inhibition of at least two processes - actin remodeling, calcium signaling, and/or MAP kinase signaling - is necessary for *Y. pestis* to inhibit degranulation [15,16]. As described above, YopE and YopH inhibit actin remodeling and calcium signaling, respectively, and have the largest individual impact on the release of granules. However, both require the other to completely inhibit degranulation, or the presence of YopJ. YopJ is an acetyltransferase that specifically acetylates the active sites of multiple MAP kinase (MAPK) proteins, inhibiting their function by preventing active site phosphorylation [17,18]. While YopJ fails to inhibit degranulation on its own, in concert with either YopH or YopE, degranulation is significantly lower than when only one Yop effector is present. Moreover, as YpkA also targets Rho/Rac GTPases and actin dynamics, it can also act cooperatively with YopH, but not YopE or YopJ, to inhibit degranulation [15,16]. Inhibition of degranulation thereby promotes the survival of extracellular *Y. pestis* that are actively inhibiting phagocytosis.

## Preventing the production of inflammatory mediators

Upon activation, neutrophils can initiate the inflammatory cascade through the rapid release of inflammatory lipids like leukotriene B4 (LTB_4_), a potent chemoattractant and immune cell activator [19]. Synthesis of LTB_4_ results when neutrophils encounter two signals that independently trigger the MAPK signaling pathway and calcium flux, both of which are required for the activation of the enzymes cPLA and 5-LO that mediate synthesis of arachidonic acid and LTB_4_, respectively [20]. Inhibition of either pathway is sufficient to block LTB_4_ synthesis. As the infection progresses, MAPK signaling and NF-κB activation will also result in neutrophil release of pro-inflammatory cytokines to further recruit and activate bystander cells. Given that MAPK signaling is key to both of these responses, blocking this pathway by YopJ acetylation of TAK1 and downstream kinases is sufficient for *Y. pestis* to inhibit both LTB_4_ synthesis and IL-8 expression by neutrophils [17,21,22]. By inhibiting calcium signaling, YopH also inhibits cPLA and 5-LO activation and LTB_4_ synthesis in neutrophils [22]. Rho/Rac inactivation by YopE or YpkA can also block LTB_4_ synthesis [22]. Finally, YopM, which inhibits pyrin-mediated inflammasome activation in macrophages [23], extends the lifespan of neutrophils by inhibiting pyroptosis and IL-1β release [24]. Together, these mechanisms effectively silence neutrophil inflammatory signaling, thereby limiting activation of bystander cells and the recruitment of circulating immune cells.

## Blocking extracellular vesicle biogenesis

Extracellular vesicles (EVs) are produced by all cells including neutrophils. Small EVs (historically referred to as exosomes) are formed from the inward budding of late endosomal membranes, known as multivesicular bodies (MVBs). MVB biogenesis results in the packaging of lipids, proteins, and small RNAs within the EVs. Cargo sorting and selection is mediated through the coordinated actions of the ESCRT pathway and tetraspanin proteins (e.g., CD63 and CD81) [25]. Release of these small EVs is mediated by MVB fusion to the plasma membrane, which relies on SNARE proteins and small GTPAses (e.g., Rab27). Similarly, the biogenesis of large EVs (historically referred to as microvesicles or ectosomes) is also primarily regulated by the ESCRT pathway and small GTPases but originate from the outward budding of the cellular plasma membrane [25]. Large EV cargo is largely representative of the macromolecule composition of the cellular cytosol. Importantly, in response to inflammatory stimuli, neutrophils increase EV release and significantly alter the cargo packaged within EVs, enriching for antimicrobial peptides and inflammatory mediators [26,27]. Released EVs will interact with other cells to facilitate intercellular communication and pathogen clearance [28]. Since EV biogenesis and release is largely mediated by changes in calcium flux and cytoskeletal dynamics, *Y. pestis* inhibition of these pathways significantly impacts EV biogenesis by human neutrophils [29]. Specifically, YopE and YopH both play a key role in suppressing EV biogenesis, likely through direct manipulation of cytoskeletal dynamics [1,29]. YopH further contributes to limiting vesicle release by suppressing calcium dependent mediated fusion with the plasma membrane. Interestingly, YopK has also been shown to contribute to the disruption of EV biogenesis [29]. YopK is known to play a critical role in controlling the kinetics of effector translocation through the T3SS [30], but it remains unclear if YopK has other functions within host cells. Regardless, YopK independently alters neutrophil EV production and works cooperatively with YopE and YopH to inhibit EV biogenesis [29]. Together, the coordinated activity of these three effectors significantly alters the proteins, and likely other cargo, packaged within EVs, directly limiting the antimicrobial potential and EV-mediated immune cell recruitment and activation, which benefits bacterial survival [29].
